# Pain in head and neck cancer survivors in South Africa: A cross-sectional study

**DOI:** 10.4102/jcmsa.v2i1.56

**Published:** 2024-05-10

**Authors:** Terral Patel, Nicholas Fung, Lauren Gardiner, Kelly E. Daniels, Nathan Lu, Rene Krause, Julie Wetter, Gerrit Viljoen, Johannes J. Fagan

**Affiliations:** 1 Department of Otolaryngology, University of Pittsburgh Medical Center, Pittsburgh, United States of America; 2 Division of Interdisciplinary Palliative Care and Medicine, University of Cape Town, Cape Town, South Africa; 3 Department of Radiation Oncology, University of Cape Town, Cape Town, South Africa; 4 Division of Otolaryngology, University of Cape Town, Cape Town, South Africa

**Keywords:** head and neck cancer, pain, quality of life, opioid, patient reported outcome measure, survivorship

## Abstract

**Background:**

Pain is often a significant symptom in patients with head and neck cancer (HNC). The objective of this study was to determine the prevalence of pain, factors limiting effective pain management, and whether differences in pain management exist between the public and private health sectors in South Africa in HNC patients after they have completed treatment.

**Methods:**

A cross-sectional survey using the Brief Pain Inventory was performed on adults over the age of 18 years treated for HNC of any subsite in both a public and private hospital in Cape Town, South Africa.

**Results:**

Overall pain scores indicated mild levels of pain and impact on daily activities. The domains of life most affected by pain were general activity, sleep and mood. There was no difference in pain scores between public hospital patients versus those with private insurance. Less than half of the patients reported receiving all their necessary medications. Most of the postoperative pain was managed with paracetamol, tramadol and ibuprofen, with only 12.8% of patients using morphine.

**Conclusion:**

This study provides a survey of pain experiences in HNC patients in a middle-income country. Insurance status did not significantly impact pain experiences, indicating equitable access to pain management resources. However, the overall utilisation of morphine and medications used to treat neuropathic pain is low. Further research is needed into the complex factors associated with pain in HNC patients.

**Contribution:**

This article is an example of a standard clinical study on patient-reported outcome measures.

## Introduction

According to the Global Cancer Observatory statistics and estimates, head and neck squamous cell carcinoma (HNSCC) is the seventh most common cancer globally, with 890 000 new cases and 450 000 deaths per year. The incidence is rising in many countries and particularly in younger patients because of human papillomavirus (HPV), with a predicted 30% rise in incidence of HNSCC by 2030.^1,2^ The reported 3-year survival in the United States (US) has improved over time from 52% for those diagnosed between 1973 and 1980 to 70% to those diagnosed between 2011 and 2014.^3^ The rising incidence of HNSCC as well as improved survival will inevitably lead to a growing population of cancer survivors suffering from post-treatment symptoms.

Both acute and chronic pain management are pressing issues for patients treated for head and neck cancer (HNC). The prevalence of HNC patients who report post-treatment chronic pain ranges from 39% following curative treatment to up to 66% in advanced, metastatic or terminal disease.^4^ Moderate-to-severe pain has a prevalence of 38% among patients with any form of cancer after treatment.^4^ Following dysphagia and xerostomia, pain ranks as the third most important treatment-related effect in these patients.^5^ Pain is a well-recognised sequela of HNC and involves a complex relationship between physical, emotional, social and spiritual factors. Pain can have both physiological and psychological consequences for both the patient and their loved ones. In addition, understanding it in patients can be very difficult because of the inability to measure pain objectively.^6^ In a cohort study of patients with HNC, 46% of those with pain were more likely to report fair, poor or very poor overall quality of life compared to 12% of those without pain. Patients who reported pain were also more likely to report issues related to anxiety, appearance, recreation, mood, shoulder dysfunction, general activity, chewing and swallowing.^5^

Two-thirds of oral and pharyngeal cancers occur in developing countries; however, there is a paucity of data available on the prevalence of pain in HNC survivors globally.^7^ The World Health Organization (WHO) recommends morphine as the primary analgesic for the treatment of moderate-to-severe pain. Yet, approximately 5.5 million terminal cancer patients globally have untreated or undertreated moderate-to-severe pain. Despite a staggering 618% increase in global consumption of opioids, mainly morphine, between the 1991–1993 and 2011–2013 periods, morphine consumption in Africa remains low with the exception of a few countries.^8,9^

Multiple barriers in the South African health system prevent adequate access to opioids and effective analgesia, contributing to inadequate treatment of pain in cancer patients. These barriers include restrictive laws controlling schedule 6 and 7 medications, stigmatisation of opioids, shortage of adequately trained prescribers and high out-of-pocket expenditures for patients.^9^ South African has both a public and a private healthcare system. A great disparity exists between the two systems, with only 16% (8 million) of the population having access to private medical insurance yet receiving care from 70% of healthcare professionals who work full-time in the private sector. Forty million uninsured South Africans are dependent on a national public healthcare system, which in many instances is dysfunctional because of underfunding, mismanagement and neglect.^10,11^

The objective of this study was to determine the prevalence and character of pain in HNC patients after they have completed curative-intent cancer treatment in South Africa, a middle-income country (MIC). In addition, we aimed to explore the variables influencing the prevalence of pain, limiting factors of effective pain management, the possible effect of pain on employment and return to work, and the differences in pain and pain management in the public and private health sectors. We hypothesised that patients who are uninsured and unemployed would have higher pain scores and lower opioid use.

## Methods

### Study design

This was a cross-sectional study that included patients from both a public (Groote Schuur Hospital) and private head and neck specialist practice in Cape Town, South Africa, and was conducted between June 2022 and June 2023. Patients > 18 years of age who had completed definitive primary therapy (including surgery, chemotherapy and/or radiation) for HNC involving any subsite (e.g. nasal cavity, nasopharynx, oral cavity, oropharynx, larynx, hypopharynx, salivary gland or locoregionally advanced head and neck cutaneous cancer) were eligible for inclusion in the study. Enrollment of patients in the study took place during head and neck cancer clinic visits. All patients who were eligible for inclusion in the study and who attended the clinic that day were asked if they would like to participate in the survey. Those who agreed were appropriately consented and the survey was administered. Interpreters were used for patients who were non-English speakers.

### Data collection

**FIGURE 1-A1:**
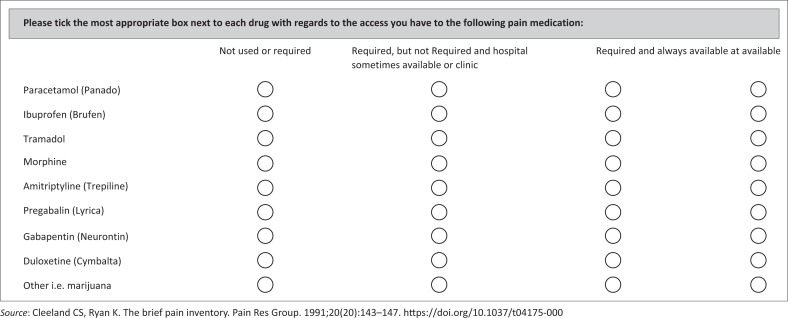
Pain survey questionnaire.

Demographic data were obtained through patient interviews and included age, gender, previous employment and medical insurance status. ‘Medical insurance’ denotes insurance that covers patients’ medical expenses. Clinicopathologic features such as primary site, stage and type of treatment were collected via paper chart review. Post-treatment pain was assessed using the Brief Pain Inventory (BPI), a validated instrument used to assess pain in cancer patients^12^ that includes an 11-point, 0 to 10 scale to assess pain as well as how pain interferes with patients’ lives. A Pain Severity Score was calculated by taking the average of the pain score at its worst, pain at its best and pain on average. A Pain Interference Score was calculated by taking the average of scores for how pain interfered with general activity, mood, work, interpersonal relationships, sleep and enjoyment of life. This tool has been translated into multiple languages and used in countries across the world including Africa.^13,14,15,16^ It has also been validated in a multicultural cohort of patients in Malaysia.^13^ The types of medications used for analgesia and their availability were part of the survey. In regard to Tramadol, it was analysed separately from other opioids such as morphine. Tramadol is a weak opioid that is readily prescribed for analgesia and is less addictive than the more potent opioids.^6^ The more potent opioids such as morphine and oxycodone were grouped together. The survey questionnaire was built using REDCap (Research Electronic Data Capture. Nashville, TN, US). The questionnaire utilised in this study is available for review as Appendix 1, Figure 1-A1.

### Statistical analysis

The Statistical Package for the Social Sciences software was used for statistical analysis (Version 29.0.0.0 for MacOS. Armonk, NY, US; IBM Corp.). Continuous variables were reported as the mean value and the standard deviation (s.d.). Categorical variables were reported as incidence and proportional percentages. The primary outcome was the prevalence and severity of pain. The secondary outcome measures were the use and availability of analgesic medications. Comparative analyses were performed using Chi-squared and Mann–Whitney testing using an alpha value of 0.05. A linear multivariable regression analysis was used to evaluate pain scores while adjusting for multiple potential confounding variables.

### Ethical considerations

This project is registered and approved by the Health Research and Ethics Committee of the Unversity of Cape Town. The IRB number is IRB00001983 and NHREC-registration number is REC-210208-007. Written consent was obtained from each study participant and an in-person interpreter was available for non-English speaking patients. Paper consent forms were kept within a locked and secure location and all data were stored on a secure online database (REDCap).

## Results

A total of 86 patients were included in the study. Mean age was 61.6 years and 61 (70.9%) were male. Although the mean age of patients in the public versus private insurance group was comparable (60.6 vs. 64.4), there were comparatively less males within the public insurance group compared to the private insurance group (67.2% vs. 81.8%). The most common primary tumour site was the oral cavity (*n* = 31, 36.0%) followed by the oropharynx (*n* = 23, 26.7%) and larynx (*n* = 22, 25.6%). Forty-six (53.5%) patients were in stages T3 or T4 and 39 (45.3%) had nodal metastases. Most patients were treated with surgery only (*n* = 23, 26.7%) or surgery with adjuvant radiation (*n* = 22, 25.6%). Trimodal therapy of surgery with chemoradiation was used in 12 (22.1%) patients and 18 (20.9%) patients were treated with primary chemoradiation alone. The mean time elapsed since completion of cancer treatment for the study sample was 31.5 months.

Twenty-two patients (25.6%) reported being members of a medical insurance scheme. The remaining 64 patients (74.4%) were reliant on the public healthcare system. There was no statistical difference in demographic and tumour characteristics by private versus public medical system status (Table 1).

**TABLE 1:** Demographic and oncologic information of study sample.

Patient characteristic	All patients (*N* = 86)				Public insurance (*n* = 64)				Medical aid (*n* = 22)				*p*
	*n*	%	Mean	s.d.	*n*	%	Mean	s.d.	*n*	%	Mean	s.d.	
**Gender**													**0.192**
Male	61	70.9	-	-	43	67.2	-	-	18	81.8	-	-	-
Female	25	29.1	-	-	21	32.8	-	-	4	18.2	-	-	-
**Age** (years)	-	-	61.6	11.5	-	-	60.6	11.5	-	-	64.4	11.2	0.194
**Primary tumour site**													**0.192**
Oral cavity	31	36.0	-	-	19	29.7	-	-	12	54.5	-	-	-
Sinonasal	2	2.3	-	-	1	1.6	-	-	1	4.5	-	-	-
Nasopharynx	1	1.2	-	-	1	1.6	-	-	0	0.0	-	-	-
Oropharynx	23	26.7	-	-	16	25.0	-	-	7	31.8	-	-	-
Larynx	22	25.6	-	-	21	32.8	-	-	1	4.5	-	-	-
Salivary gland	1	1.2	-	-	1	1.6	-	-	0	0.0	-	-	-
Skin	5	5.8	-	-	4	6.3	-	-	1	4.5	-	-	-
Other	1	1.2	-	-	1	1.6	-	-	0	0.0	-	-	-
**Tumour stage**													
T1-2	40	46.5	-	-	26	40.6	-	-	14	63.6	-	-	0.062
T3-4	46	53.5	-	-	38	59.4	-	-	8	36.4	-	-	0.084
Nodal disease	39	45.3	-	-	37	57.8	-	-	10	45.5	-	-	0.315
**Treatment modality**													
Surgery alone	23	26.7	-	-	14	21.9	-	-	9	40.9	-	-	0.082
Surgery + RT	22	25.6	-	-	19	29.7	-	-	3	13.6	-	-	0.166
Surgery + CT	1	1.2	-	-	0	0.0	-	-	1	4.5	-	-	0.256
Surgery + CRT	19	22.1	-	-	13	20.3	-	-	6	27.3	-	-	0.556
Radiation alone	2	2.3	-	-	2	3.1	-	-	0	0.0	-	-	1
Chemotherapy alone	1	1.2	-	-	1	1.6	-	-	0	0.0	-	-	1
CRT alone	18	20.9	-	-	15	23.5	-	-	3	13.6	-	-	0.544
**Time since treatment** (months)	-	-	31.5	35.0	-	-	32.3	33.4	-	-	29.4	40.0	0.546

s.d., standard deviation; *n*, sample size; RT, radiation therapy; CT, chemotherapy; CRT, chemoradiotherapy.

The results of the Brief Pain Inventory are displayed in Table 2. The mean Pain Severity Score was 2.19 (s.d.: 2.87) and the mean Pain Interference Score was 1.86 (s.d.: 2.70). There was no statistical difference in the mean Pain Severity Score or Pain Interference Score when comparing patients enrolled in public versus private healthcare systems.

**TABLE 2:** Brief pain inventory.

Patient measure	All patients				Public insurance				Medical aid				*p*
	Mean	s.d.	*n*	%	Mean	s.d.	*n*	%	Mean	s.d.	*n*	%	
**Pain scores**													
Pain Severity Score	2.19	2.87	-	-	2.43	3.06	-	-	1.50	2.15	-	-	0.344
Pain Interference Score	1.86	2.70	-	-	1.97	2.74	-	-	1.52	2.61	-	-	0.571
General activity	2.41	3.83	-	-	2.59	3.97	-	-	1.86	3.40	-	-	0.485
Mood	2.12	3.63	-	-	2.20	3.63	-	-	1.86	3.68	-	-	0.541
Work	1.74	3.19	-	-	1.89	3.31	-	-	1.32	2.84	-	-	0.471
Relationships	0.94	2.20	-	-	1.03	2.31	-	-	0.68	1.86	-	-	0.749
Sleep	2.15	3.40	-	-	2.25	3.53	-	-	1.86	3.06	-	-	0.628
Enjoyment of life	1.78	3.03	-	-	1.86	3.09	-	-	1.55	2.89	-	-	0.798
**Indirect costs**													
Employed before diagnosis	-	-	51	59.3	-	-	36	56.3	-	-	15	68.2	0.326
Employed 3 months after treatment	-	-	22	28.9	-	-	14	25.5	-	-	8	38.1	0.396
Physical type of job	-	-	34	39.5	-	-	29	80.6	-	-	5	33.3	0.002
**Medication availability**													< 0.001
Rarely use meds	-	-	22	25.6	-	-	17	26.6	-	-	5	22.7	-
I receive all I need	-	-	40	46.5	-	-	40	62.5	-	-	0	0.0	-
My medical aid covers no meds	-	-	7	8.1	-	-	2	3.1	-	-	5	22.7	-
I pay for my meds	-	-	15	17.4	-	-	3	4.7	-	-	12	54.5	-
**Medication usage**													
Paracetamol	-	-	64	74.4	-	-	48	75.0	-	-	16	72.7	0.833
Ibuprofen	-	-	33	38.4	-	-	20	31.3	-	-	13	59.1	0.021
Tramadol	-	-	41	47.7	-	-	34	53.1	-	-	7	31.8	0.137
Morphine	-	-	11	12.8	-	-	9	14.1	-	-	2	9.1	0.721
Amitriptyline	-	-	7	8.1	-	-	5	7.8	-	-	2	9.1	1
Pregabalin	-	-	5	5.8	-	-	4	6.3	-	-	1	4.5	1
Gabapentin	-	-	3	3.5	-	-	2	3.1	-	-	1	4.5	1
Duloxetine	-	-	1	1.2	-	-	1	1.6	-	-	0	0.0	1
Other	-	-	9	10.5	-	-	6	9.4	-	-	3	13.6	0.688

s.d., standard deviation.

The majority (*n* = 51, 59.3%) of patients were employed prior to their cancer diagnosis, and of patients who were 3 months or more from their treatment, 22 (28.9%) were still employed after completion of treatment. Patients reported which aspects of general life their pain interfered with. The most frequently reported were general activity (2.41, s.d.: 3.83), sleep (2.15, s.d.: 3.40) and mood (2.12, s.d.: 3.63). Patients reported that interpersonal relationships were the least impacted by their pain.

When asked about the availability of pain medications, 40 (46.5%) patients reported that they received all the medication that they needed and 22 (25.6%) reported that they rarely used pain medication. Regarding specific pain medication usage, most patients relied on paracetamol (*n* = 64, 74.4%), tramadol (*n* = 41, 47.7%) and ibuprofen (*n* = 33, 38.4%). Only 11 (12.8%) patients reported the need for morphine-like medications. Less than 10% of patients reported the use of amitriptyline, gabapentin, pregabalin or duloxetine. Only nine patients (10.5%) reported using other substances for pain relief such as marijuana. A greater proportion of private insurance versus public system patients had to pay for their pain medications (54.5% vs. 4.7%).

A multivariable analysis adjusting for patient age, gender, insurance status, employment status prior to treatment, T-stage, nodal disease status, treatment modality and time since completion of treatment was performed to determine the impact on both Pain Severity and Pain Interference Scores. However, no variables were found to be significant predictors of pain severity or pain interference.

## Discussion

This study of 86 patients diagnosed with HNC who underwent treatment in Cape Town, South Africa, revealed valuable insights into the demographics, treatment characteristics and pain experiences of this MIC population. The mean age, gender and primary tumour characteristics of our overall patient population mirrored established patterns reported in the literature.^17^ Data from an African cancer registry shows that in Africa, there is a male predominance in the incidence of HNC with a 1.8:1 ratio. In addition, the incidence ratio greatly increases for patients greater than 55 years of age.^18^ The utilisation of the Brief Pain Inventory made it possible to capture a comprehensive view of pain experiences among participants. The mean Pain Severity Score and Pain Interference Score indicated mild levels of pain (2.19; s.d.: 2.87) and impact on daily activities (1.86; s.d.: 2.70). The authors were surprised by the overall mild level of pain reported by the study population. Interestingly, no significant disparities in pain severity or interference were identified based on public versus private healthcare status. The aspects of life most affected by pain were general activity, sleep and mood, whereas relationships were least affected.

Studies of the employment status of treated HNC patients in high-income countries report rates of discontinuing work of 34% – 53%.^19,20^ Others reported that 67% – 83% of employed patients under the age of 65 years returned to work following diagnosis and treatment and 48% had reduced their workload.^19,20^ A scoping review of return to work in HNC including studies from multiple countries in North America, Europe and Asia found a very wide range of patient’s ability to return to work (37% – 92%). They stated that individual factors related to psychosocial impact and treatment toxicities were the most critical determinants of return to work.^21^ Another study found that unemployed patients report higher rates of dysphagia and dysarthria and were associated with higher levels of depression and anxiety.^22^ The economic cost because of lost employment of patients treated for HNC in our MIC patient population was greater than that reported in HICs, as only 28.9% remained employed after treatment compared to 59.3% prior to treatment. There was no difference between patients in the public and private health systems.

Multivariable analysis considering various factors including age, gender, insurance status, employment, tumour characteristics and treatment modalities did not reveal any significant predictors of pain severity or impact on daily activities. This may be because of our small sample size, but it underscores the complex and subjective nature of pain experience in HNC. Most importantly, it demonstrates that it is difficult to predict and anticipate which patients will be most affected by pain, and that pain management must be tailored to the individual patient.

Availability of medication appears to be an area for improvement in the patient population studied, as less than half of patients reported receiving all their necessary medications (54.5%). A much higher proportion of patients with private medical insurance had to pay for their medications (54.5% vs. 4.7%), indicating that many insurance plans did not cover pain morbidity associated with HNC treatment.

The most common opioid used for analgesia was tramadol. The other most common medications were over-the-counter non-opiate medications such as paracetamol and ibuprofen. Only 11 out of 86 (12.8%) patients reported using morphine-like medications. This is in line with low morphine use in lower- and middle-income countries and is in stark contrast to comparable patients in high-income countries, including the US.^10^ Another interesting finding was the low utilisation of amitriptyline, duloxetine, gabapentin and pregabalin. Neuropathic pain is common in HNC patients and responds better to α2δ-ligands and anti-depressants such as these.^6^ The low utilisation of these medications suggests a possible underdiagnosis of neuropathic pain. Consequently, we recommend assessing and re-assessing pain throughout the treatment period to appropriately modify pain management strategies.

While this study has provided some insights into pain in patients with HNC, several limitations should be acknowledged that may influence the interpretation of our findings. The study included a sample of 86 patients from a specific geographic region and medical centres. This relatively small sample size might therefore not fully represent the diversity of patients with HNC across different demographics, socioeconomic backgrounds and healthcare settings, thus limiting the generalisability of our findings to a broader population. In addition, although not statistically significant, there was a comparatively limited number of females within the private insurance group compared to the public insurance group. This limits our ability to ascertain differences in pain experience between males and females. The cross-sectional design of this study captured pain experiences and associated factors at a single point in time. This limits our ability to establish causal relationships between pain and the various demographic, clinical and treatment-related variables examined. As a result of the retrospective nature of this study, there may be a degree of recall bias, which varies from patient to patient based on how far out they are from primary treatment. A longitudinal study design would provide more robust insights into the dynamic nature of pain over the course of treatment and survivorship. In addition, the reliance on self-reported measures, such as pain severity and interference, introduces the potential for subjectivity. Patients might interpret and report their pain experiences differently, potentially leading to an underestimation or overestimation of pain levels. This pain interpretation is known to be culturally influenced. The study sample consisted of a multicultural group of patients; however, only the English version of the BPI was administered with the use of an interpreter for non-English speaking patients. This may have influenced the results and further efforts to translate the tool into Afrikaans and administer it to patients across a wider geographic range and would yield more generalisable results.

## Conclusion

This study provides an initial survey of pain experiences in HNC patients in a MIC setting. The diversity in patient demographics, tumour characteristics, treatment and pain management strategies highlight the need for individualised care of HNC patients. Insurance status did not significantly impact pain experiences, indicating equitable access to pain management resources, although privately insured patients were more likely to have to pay for medication. The main staples of analgesic medications included paracetamol, tramadol and ibuprofen. There may be an opportunity for greater utilisation of morphine-like medications and medications used to treat neuropathic pain. Further research is warranted to delve into the nuanced interactions between pain, treatment and patient-reported outcomes in HNC care.

## References

[1] Sung H, Ferlay J, Siegel RL, et al. Global cancer statistics 2020: GLOBOCAN estimates of incidence and mortality worldwide for 36 cancers in 185 countries. CA Cancer J Clin. 2021;71(3):209–249. 10.3322/caac.21660 33538338

[2] Barsouk A, Aluru JS, Rawla P, Saginala K, Barsouk A. Epidemiology, risk factors, and prevention of head and neck squamous cell carcinoma. Med Sci. 2023;11(2):42. 10.3390/medsci11020042 PMC10304137 37367741

[3] Cheraghlou S, Schettino A, Zogg CK, Judson BL. Changing prognosis of oral cancer: An analysis of survival and treatment between 1973 and 2014. Laryngoscope. 2018;128(12):2762–2769. 10.1002/lary.27315 30194691

[4] Van Den Beuken-Van MH, Hochstenbach LM, Joosten EA, Tjan-Heijnen VC, Janssen DJ. Update on prevalence of pain in patients with cancer: systematic review and meta-analysis. J Pain Symptom Manage. 2016;51(6):1070.e9–1090.e9. 10.1016/j.jpainsymman.2015.12.340 27112310

[5] Cramer JD, Johnson JT, Nilsen ML. Pain in head and neck cancer survivors: prevalence, predictors, and quality-of-life impact. Otolaryngol Neck Surg. 2018;159(5):853–858. 10.1177/0194599818783964 29943677

[6] Blanchard C, Chetty S, Ganca L, Gwyther L, Hodgson E. Guide to the treatment of cancer pain in South Africa. Pretoria: Medspec Publishing; 2015.

[7] Warnakulasuriya S. Global epidemiology of oral and oropharyngeal cancer. Oral Oncol. 2009;45(4–5):309–316. 10.1016/j.oraloncology.2008.06.002 18804401

[8] World Health Organization. WHO model lists of essential medicines [homepage on the Internet]. Geneva: World Health Organization; 2017 [cited n.d.]. Available from: https://www.who.int/groups/expert-committee-onselection-and-use-of-essential-medicines/essentialmedicines-lists

[9] Board INC. Availability of internationally controlled drugs: ensuring adequate access for medical and scientific purposes: Indispensable, adequately available and not unduly restricted. New York, NY: UN; 2016.

[10] Namisango E, Allsop MJ, Powell RA, et al. Investigation of the practices, legislation, supply chain, and regulation of opioids for clinical pain management in Southern Africa: a multi-sectoral, cross-national, mixed methods study. J Pain Symptom Manage. 2018;55(3):851–863. 10.1016/j.jpainsymman.2017.11.010 29155288

[11] Mayosi BM, Benatar SR. Health and health care in South Africa – 20 years after Mandela. N Engl J Med. 2014;371(14):1344–1353. 10.1056/NEJMsr1405012 25265493

[12] Cleeland CS, Ryan K. The brief pain inventory. Pain Res Group. 1991;20(20):143–147. 10.1037/t04175-000

[13] Kiu DKL, Lee ZFD, Voon PJ. Exploration of patient-related barriers to Effective Cancer Pain Management in a diverse multicultural developing country. J Pain Symptom Manage. 2021;62(1):75–80. 10.1016/j.jpainsymman.2020.11.011 33197524

[14] Parker R, Jelsma J, Stein DJ. Pain in amaXhosa women living with HIV/AIDS: Translation and validation of the Brief Pain Inventory–Xhosa. J Pain Symptom Manage. 2016;51(1):126.e2–132.e2. 10.1016/j.jpainsymman.2015.08.004 26344550

[15] Tan G, Jensen MP, Thornby JI, Shanti BF. Validation of the Brief Pain Inventory for chronic nonmalignant pain. J Pain. 2004;5(2):133–137. 10.1016/j.jpain.2003.12.005 15042521

[16] Abebe AB, Ayele TA, Miller J. Evaluating the validity of the Amharic Brief Pain Inventory among people with chronic primary musculoskeletal pain in Ethiopia. BMC Musculoskelet Disord. 2022;23(1):875. 10.1186/s12891-022-05833-5 36131337 PMC9490988

[17] Adeola HA, Afrogheh AH, Hille JJ. The burden of head and neck cancer in Africa: The status quo and research prospects. S Afr Dent J. 2018;73(8):477–488. 10.17159/2519-0105/2018/v73no8a1

[18] Auguste A, Gathere S, Pinheiro PS, et al. Heterogeneity in head and neck cancer incidence among black populations from Africa, the Caribbean and the USA: Analysis of cancer registry data by the AC3. Cancer Epidemiol. 2021;75:102053. 10.1016/j.canep.2021.102053 34743058 PMC8627451

[19] Verdonck-de Leeuw IM, Van Bleek WJ, Leemans CR, De Bree R. Employment and return to work in head and neck cancer survivors. Oral Oncol. 2010;46(1):56–60. 10.1016/j.oraloncology.2009.11.001 20004135

[20] Giuliani M, Papadakos J, Broadhurst M, et al. The prevalence and determinants of return to work in head and neck cancer survivors. Support Care Cancer. 2019;27:539–546. 10.1007/s00520-018-4343-6 30014191

[21] Zhang Y, Wang Y, Wu A, et al. The prevalence and determinants of return to work in head and neck cancer survivors: A scoping review. J Occup Rehabil. 2023;33(3):418–431. 10.1007/s10926-022-10090-3 36689058

[22] Broemer L, Friedrich M, Wichmann G, et al. Exploratory study of functional and psychological factors associated with employment status in patients with head and neck cancer. Head Neck. 2021;43(4):1229–1241. 10.1002/hed.26595 33615608

